# Analyzing CRISPR screens in non-conventional microbes

**DOI:** 10.1093/jimb/kuad006

**Published:** 2023-03-16

**Authors:** Varun Trivedi, Adithya Ramesh, Ian Wheeldon

**Affiliations:** Department of Chemical and Environmental Engineering, University of California, Riverside, CA 92521, USA; Department of Chemical and Environmental Engineering, University of California, Riverside, CA 92521, USA; Department of Chemical and Environmental Engineering, University of California, Riverside, CA 92521, USA; Center for Industrial Biotechnology, University of California, Riverside, CA 92521, USA; Integrative Institute for Genome Biology, University of California, Riverside, CA 92521, USA

**Keywords:** Functional genomics, CRISPR screening, Non-conventional yeasts, Genetic screen analysis, Genotype–phenotype relationships

## Abstract

The multifaceted nature of CRISPR screens has propelled advancements in the field of functional genomics. Pooled CRISPR screens involve creating programmed genetic perturbations across multiple genomic sites in a pool of host cells subjected to a challenge, empowering researchers to identify genetic causes of desirable phenotypes. These genome-wide screens have been widely used in mammalian cells to discover biological mechanisms of diseases and drive the development of targeted drugs and therapeutics. Their use in non-model organisms, especially in microbes to improve bioprocessing-relevant phenotypes, has been limited. Further compounding this issue is the lack of bioinformatic algorithms for analyzing microbial screening data with high accuracy. Here, we describe the general approach and underlying principles for conducting pooled CRISPR knockout screens in non-conventional yeasts and performing downstream analysis of the screening data, while also reviewing state-of-the-art algorithms for identification of CRISPR screening outcomes. Application of pooled CRISPR screens to non-model yeasts holds considerable potential to uncover novel metabolic engineering targets and improve industrial bioproduction.

**One-Sentence Summary:**

This mini-review describes experimental and computational approaches for functional genomic screening using CRISPR technologies in non-conventional microbes.

## Graphical Abstract

**Graphical Abstract:**
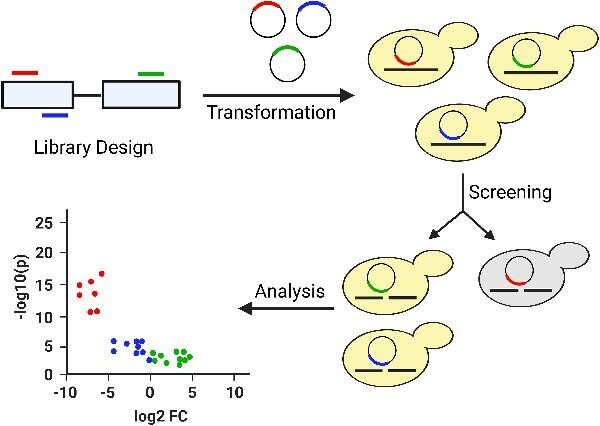
Schematic representation of the end-to-end pipeline for performing pooled CRISPR screens in microbes. Major steps include sgRNA library design, phenotypic screening, and quantitative analysis of the screening data.

## Introduction

High-throughput CRISPR screens have become a versatile tool in enabling identification of the genetic basis of various phenotypes (Doench, 2018; Hart et al., 2015; Peters et al., 2016). For instance, they have been used extensively in mammalian cancer cell lines to identify essential genes for survival, for facilitating targeted drug design, and in immunological studies to identify genes involved in various pathways in human immune cells (Aguirre et al., 2016; Meyers et al., 2017; Parnas et al., 2015; Shifrut et al., 2018; Tzelepis et al., 2016). Moreover, with the ability to target combinations of multiple genes simultaneously, CRISPR screens have made it possible to elucidate functions of poorly characterized genes via construction of gene interaction maps (Horlbeck et al., 2018). Genome-wide CRISPR screens have also been performed in bacteria and yeasts to unravel genetic hits influencing a diverse set of phenotypes, including those relevant to industrial bioproduction. Previous studies in model microbes—*Escherichia coli* and *Saccharomyces cerevisiae—*have identified essential genes as well as those required for conferring tolerance to biochemicals like isobutanol and furfural (Bao et al., 2018; Rousset et al., 2018; Wang et al., 2018). Other studies have focused on non-conventional microbes, such as the oleaginous yeast *Yarrowia lipolytica*, to identify genes essential for growth on glucose, and those important for providing tolerance to environmental stress conditions, such as low pH and high salt concentration (Ramesh et al., 2022; Schwartz et al., 2019).

CRISPR screens typically use a library of programmable single guide RNAs (sgRNAs) and a CRISPR-associated endonuclease, typically Cas9 or Cas12a, to create mutations or alter gene expression (Bock et al., 2022; Bodapati et al., 2020; Kampmann, 2018). The most common type of CRISPR screens is knockout screens where the CRISPR-Cas system generates a double-stranded break at the genomic target site, evoking native DNA repair pathways such as non-homologous end joining to create INDEL mutations that result in loss of gene function (Shalem et al., 2014; Wang et al., 2014). Besides knocking out gene function, CRISPR screens can make use of a nuclease-deactivated Cas protein to modulate transcription when fused to activator (CRISPR activation or CRISPRa) or repressor (CRISPR interference or CRISPRi) domains. These screens can be conducted in a pooled or arrayed format. Arrayed screens physically separate predefined gene perturbations, making them malleable to amalgamation with downstream -omics profiling; but they have limited throughput and are relatively expensive (Bock et al., 2022; Bodapati et al., 2020; Kampmann, 2018). On the contrary, pooled screens have a much higher throughput as they are devoid of physical separation between gene targets, making them more commonplace compared to arrayed screens, but require performing comparisons to a baseline for hit identification (Bock et al., 2022; Bodapati et al., 2020). The customizable nature of sgRNA and the ease of inducing perturbations to gene function using CRISPR-Cas systems make high-throughput CRISPR screens an effective tool for establishing genotype–phenotype relationships in both model and non-conventional organisms.

The CRISPR screening workflow comprises of a series of experimental and computational steps, ranging from host selection and library design to identification and biological interpretation of hits. In this review, we explore some of these steps in detail, with a focus on pooled CRISPR knockout screening in non-conventional yeasts. We begin by discussing the experimental design for conducting the screens, followed by bioinformatic processing of screening data. We also describe the general working principles behind the identification of screening outcomes while juxtaposing the nature of yeast and mammalian cell datasets. Lastly, we review some of the existing algorithms for analyzing CRISPR screens and discuss their performance on yeast screening datasets, with a goal of assisting researchers in choosing the most appropriate tool for analyzing their data.

## Functional Genetic Screening with Pooled CRISPR Libraries

**Fig. 1:**
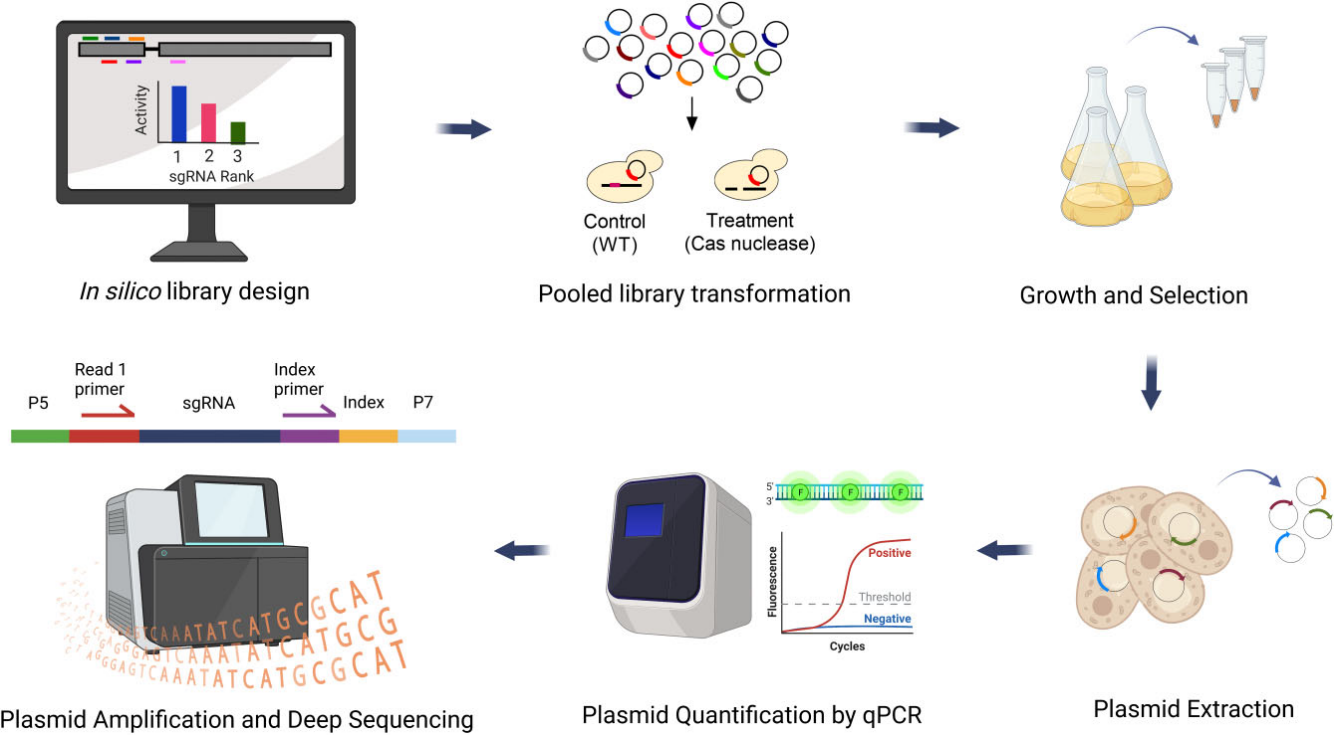
Experimental workflow for conducting pooled CRISPR knockout screens. A library of sgRNA spanning multiple genomic sites is designed, synthesized, and cloned into a plasmid backbone. The plasmid library is transformed into control and treatment host strains, and cells are cultivated for a predetermined number of days to select for significant gene knockouts. Upon completion of the screen, plasmids are extracted from the cells, quantified by qPCR, and sequenced using NGS. Figure created with BioRender.com.

A schematic representation of the experimental pipeline for performing pooled CRISPR knockout screens is depicted in Fig. 1. Since microbes exhibit different sets of desirable phenotypes, a preliminary step in CRISPR screening is the selection of an appropriate biological host for a given application. In the case of non-conventional yeasts, relevant phenotypes influencing host selection include a microorganism's natural ability to synthesize a certain bioproduct or tolerate harsh environmental conditions that may be present in industrial bioprocesses (Thorwall et al., 2020). Once a host is chosen, an sgRNA library is designed to target relevant or all protein-coding genes in the genome of the organism (although non-coding regions could also be targeted [Shukla & Huangfu, 2018]).

### sgRNA Library Design

It is well known that guide RNAs present disparities in inducing genetic edits and that guide activity is crucial to the accuracy of screening results. Highly active guides can correlate the phenotypic variations to the appropriate genomic perturbation with high accuracy, while poorly active guides may obscure gene hits. It is thus advantageous to create a library comprising a large proportion of high-activity guides to improve hit calling. High-activity libraries can be designed by picking guides based on *in silico* activity scores estimated using activity prediction algorithms. Existing software tools such as CHOPCHOP (Labun et al., 2019), CRISPRLearner (Dimauro et al., 2019), DeepCpf1 (Kim et al., 2018), and DeepGuide (Baisya et al., 2022), among others, use one or more sequence, structural, and epigenetic features of sgRNA to predict on-target activity with endonucleases, such as Cas9 and Cas12a. See Konstantakos et al., (2022) for an in-depth review on guide activity prediction and benchmarking across current tools. Despite most of the prediction algorithms being developed for model organisms, they have been reasonably effective in facilitating the design of active sgRNA libraries in non-conventional hosts relative to an unbiased approach that is blinded to *in silico* activity scores (Ramesh et al., 2022; Schwartz et al., 2019). Even when using activity prediction scores, it is advisable to design several guides targeting each gene (i.e., a high genome coverage), ensuring the presence of an active guide per gene and sufficient statistical power for hit identification—an approach that comes with a cost of increasing downstream analytical complexity. Regardless of the design strategy, it is critical to ensure that every sgRNA in the final library is (i) unique within the genome, so that off-target effects are minimized; (ii) does not target intronic regions of the coding sequence; (iii) sufficiently spaced from other sgRNA to improve diversity of target locations; and if possible, (iv) located within 5–65% of each coding sequence to maximize the chances of a gene knockout resulting in a non-functional protein (Doench et al., 2016; Ramesh & Wheeldon, 2021).

### Conducting the Screening Experiment

The designed library is synthesized as pooled single-stranded oligos on a DNA microchip that are cloned into a plasmid vector, resulting in a library of plasmids. This plasmid library is amplified by transforming it into *E. coli* before isolation and subsequent transformation into the actual host cells for the screening experiment. Stable Cas expression in host cells is often accomplished through heterologous expression from a genomically integrated expression cassette. In addition to the sample (or treatment) strain, a control (or reference) strain is also needed so that changes in guide abundances at the end of the screen can be determined. In many cases, a strain devoid of Cas endonuclease but harboring the guide RNA library is used as a control (Hart et al., 2015; Koike-Yusa et al., 2014). Other examples of the reference conditions include the treatment sample immediately post-transformation (day 0 of the screen before gene knockouts occur) or the untransformed library (Meyers et al., 2017).

Upon transformation of the sgRNA library in the control and treatment strains, cells are allowed to proliferate until they reach confluency, and then subcultured to allow for genetic selection. At the end of the screen, the connection between genotype and phenotype is made by sequencing isolated plasmids expressing the sgRNA. That is, the fitness effect of disrupting a given gene is determined by quantifying the abundance of the sgRNA targeting the gene. To do so, the plasmid library is extracted from the treatment and control samples, the encoded guides are amplified by PCR, and the amplicon pool is sequenced using an Illumina or similar NGS platform. For accuracy of results, it is advisable to ensure sufficient depth in the sequencing run, which should be about 100 times the library size or higher for every screening replicate.

The resulting sequencing reads, the counts of which are indicative of the abundance of a given mutant in the microbial population, can be bioinformatically processed to identify genes that affect growth in the screened condition. The raw sgRNA abundances themselves cannot be used directly for accurate determination of screening outcomes, since they do not account for variability in sgRNA activity and variability in sequencing depth across samples, necessitating bioinformatic analysis to obtain significant hits from the screen.

## CRISPR Screen Data Analysis

**Fig. 2:**
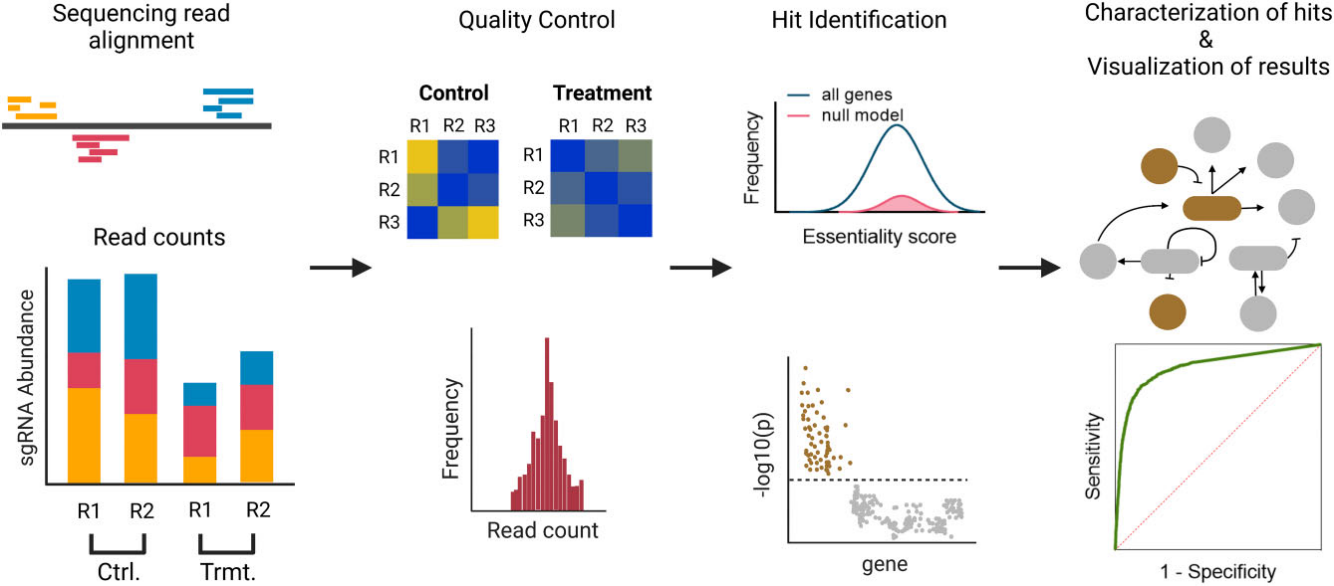
Typical steps in pooled CRISPR screen analysis and visual depiction of results. Raw sequencing reads from the screen are processed to generate sgRNA read counts, which, upon passing quality control, are used to identify significant genes in the dataset. The identified hits are characterized to elucidate underlying biological mechanisms, and screening results are visualized by making plots. Figure created with BioRender.com.

CRISPR screen analysis pipelines typically include steps for sequencing read processing, quality control, hit identification, and investigation of screening results. These steps and a typical analysis pipeline are shown in Fig. 2, and described in detail below along with the associated bioinformatic tools available for use.

### Processing Raw Sequencing Data

While analysis tools like PinAPL-Py (Spahn et al., 2017) and MAGeCK (Wang et al., 2019) can accept raw sequencing data as input and process it as part of their pipelines, most other analysis packages require sgRNA abundances or log2-fold changes as input. Consequently, raw sequencing reads from the genome-wide screen need to be modified and aligned separately before analyzing them to generate screening outcomes. This can be accomplished with a number of existing bioinformatics tools and workflows. For instance, FastQC (Andrews & Others, 2010) allows users to perform quality control on the sequencing data based on metrics such as per base sequence quality, sequence length distribution, and overrepresented sequences, among others.

If multiple screening samples and replicates were sequenced in a single run, then the reads could be demultiplexed to split the data into individual samples and replicates on the basis of sample-specific adapters. This is achieved with the help of tools such as Cutadapt (Martin, 2011), Ultraplex (Wilkins et al., 2021), or ea-utils (Aronesty, 2011). Other tools like fastp (Chen et al., 2018) or Trimmomatic (Bolger et al., 2014) could be used to trim the reads by removing the vector backbone and other miscellaneous sequences to only retain the sgRNA sequence.

In regard to mapping reads to the genome and/or the sgRNA library, available methods, include BWA-MEM2 (Vasimuddin et al., 2019), Bowtie2 (Langmead & Salzberg, 2012), or HISAT2 (Kim et al., 2019), which align NGS reads to a reference sequence by exact or approximate matching. Of these tools, Bowtie2 is most widely used for read mapping in CRISPR screen analysis. The read alignment information is used to compute the read count (i.e., abundance) of each sgRNA across samples.

For CRISPR screens in non-model yeasts like *Y. lipolytica*, the tools Cutadapt and Trimmomatic have been found to be suitable for demultiplexing and trimming sequencing reads, respectively (Baisya et al., 2022; Ramesh et al., 2022). Similarly, a combination of Bowtie2 and naive exact matching has been shown to perform reasonably well in aligning reads, especially due to the ability of Bowtie2 to account for mismatches during alignment, that mainly stem from sequencing errors.

### Quality Control of the Screening Data

Before using the read counts for further analysis, it is essential to assess the quality of experimental data, for example, by determining pairwise replicate correlation coefficients per sample and examining the sgRNA abundance distribution in the original library. This is done to ensure authenticity of screening results and reduce spurious hit predictions. High correlation values (e.g., Pearson's coefficient >0.7) indicate consistency between biological replicates. Upon passing this quality check, raw sgRNA read counts from control and treatment samples are provided to one or more CRISPR screen analysis methods that employ statistical approaches to identify significant genes in the screen.

### Identifying Screen Hits

Most methods normalize the raw sgRNA abundances to account for varying sequencing depths across samples and ensure a fair comparison between controls and treatments. These normalized abundances are used directly or converted to log2-fold change to estimate gene essentiality scores, predominantly using Bayesian principles.

The genome-wide library contains sgRNA with variable activity; failure to account for this variability could lead to inaccurate predictions of screening results. High-activity sgRNA should thus have a greater influence in determining gene essentiality compared to low-activity sgRNA. A common strategy to infer sgRNA activity involves screening across multiple conditions and applying probability-based approaches to make a prediction (Allen et al., 2019; Li et al., 2015). Alternatively, guide RNA activity can be determined experimentally by screening in an additional treatment sample containing a knockout of the native DNA repair mechanism. The activity of sgRNA can then be estimated as the log2-fold change in sgRNA abundance in the knockout-containing strain (in the presence of the Cas endonuclease) to that in the control strain (Ramesh et al., 2022; Schwartz et al., 2019). Using this approach not only improves the reliability of the activity estimate, but also avoids the need to screen across multiple conditions (substantially reducing the size of the experiment), although knockout of DNA repair may not always be viable for all organisms.

Once essentiality scores have been computed, a statistical test for every gene to determine whether it belongs to a ‘‘null’’ population of scores (i.e., population of essentiality scores of non-essential genes) is typically conducted, thus resulting in a *p*-value for the essentiality of each gene. The *p*-value is further corrected for multiple comparisons (typically using FDR; Benjamini & Hochberg, 1995), and genes having a corrected *p*-value lower than a predetermined threshold are deemed as significant hits or essential genes.

### Selecting a Null Model for Significance Testing

A suitable choice for the ‘‘null’’ population depends on the host organism used to generate the screening data. Ideally, the null population is representative of the behavior of non-essential genes in the screen. For mammalian cells, the non-essential gene population overlaps well with the population of negative control sgRNA, and as a result, the negative control population serves as a suitable null model. On the other hand, non-conventional yeast datasets, in our experience, have a non-essential gene population that is relatively distant from the population of negative control sgRNA (Ramesh et al., 2022). Using negative controls to create the null population would thereby result in a large number of false essentiality predictions, prompting the use of putative non-essential genes to create the null population.

While negative control sgRNAs do not make knockouts in the genome, knockouts produced by targeting sgRNA result in growth defects and a corresponding drop in the targeting sgRNA abundance compared to the control sample. The proximity between the negative control sgRNA and non-essential gene populations thus depends on the ability of host cells to stem these growth defects. Non-conventional yeasts lack this ability to suppress growth defects, likely due to the absence of multiple gene copies and alternate splicing mechanisms. This is in contrast to the case of mammalian cells, which exhibit polyploidy and undergo alternate splicing of genes, presumably suppressing growth defects and causing the non-essential gene population to overlap well with the negative control population.

### Investigation of Screening Results

After identifying significant genes from a screen, the next step is to elucidate their biological importance. Databases such as UnitProt (UniProt Consortium, 2015) and Pfam (Sonnhammer et al., 1997) can be used to investigate protein functions of known genes. In addition, analyses like gene ontology-enrichment test (Ashburner et al., 2000) and GSEA (Subramanian et al., 2005) could be performed to identify biological pathways relevant to the significant hits. Since non-model organisms have a considerable number of unannotated genes, these could be investigated by performing BLAST (Altschul et al., 1990) against proteomes of model organisms or more rigorously by experimentation. Finally, screening results can be visualized, for example, by plotting log2-fold changes of sgRNA targeting significant genes against a backdrop of those of the entire library. Moreover, if a gold-standard set of essential genes is available, then receiver operator characteristic plots or precision–recall (PR) plots can be constructed and area under the curve can be calculated to determine accuracy of the predictions.

## Software Packages for CRISPR Screen Analysis

Here, we introduce and describe the most commonly used software packages for analyzing pooled CRISPR screens. A comparison of the tools based on some common features is provided in Table 1.

**Table 1.** Comparison of Software Packages for Analysis of Pooled CRISPR Screens

	Implement-	Quality	Experimental	Multiple	Applicable to	
Method	ation	control	sgRNA efficiency	screens	CRISPRa/i	Reference(s)
MAGeCK-VISPR	Python	Yes	No	Yes	No	Li et al. (2015)
CRISPhieRmix	R,C++	No	No	No	Yes	Daley et al. (2018)
JACKS	Python	No	No	Yes	No	Allen et al. (2019)
ACE	R	No	No	Yes	No	Hutton et al. (2021)
BAGEL2	Python	Yes	No	No	No	Kim & Hart (2021)
acCRISPR	Python	No	Yes	No	No	Ramesh et al. (2022)

### MAGeCK-VISPR

MAGeCK-VISPR is an end-to-end workflow for quality control, analysis, and visualization of CRISPR screens (Li et al., 2015). The analysis is carried out by an expectation–maximization algorithm that takes raw sgRNA counts from multiple screening conditions as input and uses them to iteratively compute gene essentiality across conditions and sgRNA activity. Read counts are modeled as a negative binomial distribution, and a generalized linear model is used to deconvolute gene effects from multiple screens. Although shown to be robust in making predictions for mammalian cancer cell lines, the method inaccurately estimates sgRNA activity for datasets from non-conventional yeasts, which leads to erroneous predictions for gene essentiality (Ramesh et al., 2022).

### CRISPhieRmix

Originally designed to analyze CRISPRa and CRISPRi screens, CRISPhieRmix can also be applied to knockout screens (Daley et al., 2018). The method requires log2-fold changes of sgRNA as input and fits that data to a hierarchical mixture distribution, constituting a broad-tailed null distribution (to account for asymmetry in the screening data) and an alternative distribution. This model is used to compute and return the posterior probability of belonging to the alternative distribution for each gene, marginalized over all possible mixture distributions of sgRNA targeting essential genes. Since CRISPhieRmix uses the negative control population to form the null distribution, it performs well on screening data from human cancer cells but has been found to result in an excessive number of false positives for screening datasets in the yeast *Y. lipolytica* (Ramesh et al., 2022).

### JACKS

JACKS is a Bayesian method that processes data from multiple screens simultaneously to improve the modeling of sgRNA activity and hence, estimation of condition-dependent gene essentiality (Allen et al., 2019). The method starts out by assuming Gaussian priors for gene essentiality scores and sgRNA efficacies. It further uses raw sgRNA counts as input to compute log2-fold changes that constitute the likelihood function. The final values of sgRNA activity and gene essentiality per condition are inferred from their respective posteriors and determined using variational inference. Like MAGeCK-MLE, JACKS effectively identifies essential genes in human datasets, but has been shown to fall short of correctly classifying essential genes in non-conventional yeasts like *Y. lipolytica*, mainly due to its inability to make accurate sgRNA activity inferences (Ramesh et al., 2022).

### BAGEL2

Developed as an updated version of BAGEL (Hart & Moffat, 2016), this method uses information from gold-standard sets of essential and non-essential genes to infer essentiality of every gene in the screening dataset, via calculation of a Bayes factor corrected for off-target effects (Kim & Hart, 2021). BAGEL2 accounts for copy number effect using the tool CRISPRcleanR (Iorio et al., 2018). Additionally, it determines the quality of each screening replicate by computing a quality score based on log-fold change of sgRNA targeting reference essential and non-essential genes. Since gold-standard sets of essential and non-essential genes may not always be available, as is the case with most non-model organisms, this method may have limited cross-species applicability at present.

### ACE

ACE is a probabilistic method with the ability to predict differential gene essentiality between samples, in addition to absolute essentiality (Hutton et al., 2021). The method does this using the sgRNA abundance in the untransformed library, along with initial and final abundances from each screening sample, which are all modeled as Poisson distributions and help define the likelihood function. Knockout efficiency of sgRNA is computed using a logistic regression model, assuming that it depends on the GC content of each guide sequence. Finally, ACE estimates gene essentiality and the logistic regression coefficients iteratively using maximum-likelihood estimation and determines gene significance from separate likelihood ratio tests for absolute and differential essentiality. Thus far, this analysis package has primarily been used to successfully identify gene essentiality in mammalian cancer cell lines (Hutton et al., 2021).

### acCRISPR

acCRISPR is an activity-correction method that improves CRISPR screening outcomes by optimizing sgRNA library activity (Ramesh et al., 2022). The method uses experimental sgRNA efficiency profiles, obtained by knocking out the dominant host DNA repair mechanism (such as non-homologous end joining by deletion of *ku70* gene), to remove low-activity sgRNA from the analysis and correct screening outcomes based on an activity threshold, by calculating an ac-coefficient (given as the product of sgRNA activity threshold and library coverage). In the absence of experimental activity values, acCRISPR can utilize predicted activity scores for the library, if available. Gene essentiality is determined by testing against a null distribution, created using sgRNA targeting putative non-essential genes, which makes acCRISPR a suitable method for analyzing screens in non-model yeasts. This method has been shown to accurately call essential genes and genes important for environmental stress tolerance in the oleaginous yeast *Y. lipolytica* (Ramesh et al., 2022).

## Conclusions and Perspectives

Pooled CRISPR screens have shown great promise in facilitating biological discovery by enabling identification of genetic signatures for known and novel phenotypes. Although CRISPR screens have been extensively used in mammalian cells to investigate disease mechanisms, their application to non-conventional microbes for improving metabolic engineering-relevant phenotypes has been limited so far. This review describes the experimental and computational steps involved in conducting and analyzing CRISPR knockout screens, with a focus on approaches and methods that have been successfully deployed in non-conventional yeasts. While the integration of these steps makes for an end-to-end workflow, there are several considerations that one needs to be mindful of in the entire process.

The ability of the sgRNA library to produce genetic knockouts, for instance, plays a pivotal role in determining the effectiveness of a screen. Accordingly, libraries should be formulated to include as many high-activity guides as possible. This could be achieved in part by using activity scores obtained from sgRNA activity prediction tools to inform library design. In the absence of accurate activity predictions, as is often the case with most non-model organisms, it is advisable to create a library having high genome coverage to ensure sufficient statistical power in evaluating screening outcomes.

Another key aspect of improving screen design and analysis is the successful delineation of sgRNA activity profiles in the context of the screen itself. While predicted activity scores may be readily available, sgRNA efficiencies are susceptible to variation in the screening environment, warranting this extra measurement. Such activity profiles can be derived experimentally or by modeling single or multiple screens. This additional data can be leveraged to diminish the influence of low-activity sgRNA in estimating gene effects, thereby enhancing the accuracy of hit identification. Other considerations for optimizing experimental design of the screen include using an adequate number of biological replicates, ensuring high library representation at the start of the screen, and sequencing the library at a sufficient depth.

Overall, CRISPR knockout screening in non-conventional microbes is an evolving tool that could be harnessed to investigate biological mechanisms and thus decode the genetics of the host organism. In addition to knockout screening, future studies should focus on knockdown and activation screens (CRISPRi and CRISPRa, respectively), promoting discovery of gene function and establishment of novel genotype–phenotype relationships. These biological findings would further improve host genetic engineering, drive enhancement of desirable phenotypes, and consequently improve the feasibility of industrial bioprocesses.
